# Aberrant Dyskerin Expression Is Related to Proliferation and Poor Survival in Endometrial Cancer

**DOI:** 10.3390/cancers13020273

**Published:** 2021-01-13

**Authors:** Rafah Alnafakh, Gabriele Saretzki, Angela Midgley, James Flynn, Areege M. Kamal, Lucy Dobson, Purushothaman Natarajan, Helen Stringfellow, Pierre Martin-Hirsch, Shandya B. DeCruze, Sarah E. Coupland, Dharani K. Hapangama

**Affiliations:** 1 Liverpool Women’s Hospital NHS Foundation Trust, Member of Liverpool Health Partners, Liverpool L8 7SS, UK; R.A.A.Alnafakh@liverpool.ac.uk (R.A.); lucy.dobson@liverpool.ac.uk (L.D.); Puru.Natarajan@lwh.nhs.uk (P.N.); Bridget.Decruze@lwh.nhs.uk (S.B.D.); 2 Department of Women’s and Children’s Health, Institute of Life Course and Medical Sciences, University of Liverpool, Member of Liverpool Health Partners, Liverpool L8 7SS, UK; areegekamal@gmail.com; 3 Department of Pathology, Al-Hilla Teaching Hospital, Babil, Iraq; 4 Biosciences Institute, Campus for Ageing and Vitality, Newcastle University, Newcastle upon Tyne NE4 5PL, UK; gabriele.saretzki@newcastle.ac.uk; 5 Experimental Arthritis Treatment Centre for Children, Institute in the Park, Department of Women’s and Children’s Health, University of Liverpool, Liverpool L12 2AP, UK; Angela.Midgley@liverpool.ac.uk; 6 Illumina Inc., San Diego, CA 92122, USA; jflynn@illumina.com; 7 Pathology Department, Oncology Teaching Hospital, Baghdad Medical City, Baghdad, Iraq; 8 Lancashire Teaching Hospital NHS Trust, Preston PR2 9HT, UK; helen.stringfellow@lthtr.nhs.uk (H.S.); Pierre.Martin-Hirsch@lthtr.nhs.uk (P.M.-H.); 9 Molecular and Clinical Cancer Medicine, Institute of Systems, Molecular and Integrative Biology, University of Liverpool, Liverpool L7 8TX, UK; s.e.coupland@liverpool.ac.uk

**Keywords:** dyskerin, *DKC1*, endometrial cancer, telomerase, proliferation, telomeres

## Abstract

**Simple Summary:**

Telomeres are the protective caps at the ends of chromosomes, and they are maintained by an enzyme called telomerase. Telomerase activity allows rapid reproduction of the cells (proliferation) of the lining of the womb (endometrium). Telomerase levels are high in cancers in general, including in endometrial cancer. Dyskerin is one of the main components of the telomerase enzyme. While the other main components of telomerase have been studied in endometrial cancer, there are no previous studies on dyskerin in the endometrium. Our study shows that dyskerin levels are significantly lower in endometrial cancer and levels are linked to the survival of women. Experimentally increasing dyskerin protein in endometrial cells in the laboratory reduces the rate of cell proliferation. Consequently, we propose that dyskerin may be a regulator of endometrial cancer cell proliferation, and further studies are required to test if it can be targeted to develop new therapies for endometrial cancer.

**Abstract:**

Dyskerin is a core-component of the telomerase holo-enzyme, which elongates telomeres. Telomerase is involved in endometrial epithelial cell proliferation. Most endometrial cancers (ECs) have high telomerase activity; however, dyskerin expression in human healthy endometrium or in endometrial pathologies has not been investigated yet. We aimed to examine the expression, prognostic relevance, and functional role of dyskerin in human EC. Endometrial samples from a cohort of 175 women were examined with immunohistochemistry, immunoblotting, and qPCR. The EC cells were transfected with Myc-DDK-DKC1 plasmid and the effect of dyskerin overexpression on EC cell proliferation was assessed by flow cytometry. Human endometrium expresses dyskerin (*DKC1*) and dyskerin protein levels are significantly reduced in ECs when compared with healthy postmenopausal endometrium. Low dyskerin immunoscores were potentially associated with worse outcomes, suggesting a possible prognostic relevance. Cancer Genome Atlas (TCGA) ECs dataset (*n* = 589) was also interrogated. The TCGA dataset further confirmed changes in *DKC1* expression in EC with prognostic significance. Transient dyskerin overexpression had a negative effect on EC cell proliferation. Our data demonstrates a role for dyskerin in normal endometrium for the first time and confirms aberrant expression with possible prognostic relevance in EC. Interventions aimed at modulating dyskerin levels may provide novel therapeutic options in EC.

## 1. Introduction

Telomeres are specialised nucleoprotein complexes consisting of tandem repeats of TTAGGG and associated specific shelterin proteins [1]. They prevent chromosomal ends from being identified as DNA damage and protect them from degradation and end to end fusion [2,3]. With each round of cell division, telomeric DNA is lost due to the end replication problem as well as oxidative stress [4,5]. In mitotic cells, critical shortening of telomeres induces apoptosis and senescence [6]. Telomerase is a specialised reverse-transcriptase which maintains and elongates telomeres [7] and is composed of: (i) the template-containing telomerase RNA component (TERC), (ii) the catalytic component of the enzyme, human telomerase reverse transcriptase (hTERT) and (iii) the protein dyskerin as one of the main core components [8]. In most human somatic cells, telomerase activity (TA) is either undetectable or very low [9]. However, human cells with high replicative demand such as lymphocytes [10] epithelial cells [11] and tissue stem cells have active or inducible telomerase [12]. The human endometrium is a highly regenerative tissue with a dynamic TA corresponding to epithelial proliferation [13]. Most cancer cells express constitutively high TA, providing them with an indefinite proliferative ability [14].

EC is the commonest gynaecological malignancy in developed countries, with an increasing incidence [15]. In an era of decreasing cancer-related deaths reported for most other cancers, mortality due to EC is expected to increase [16]. Therefore, novel biomarkers to stratify high-risk patients for therapy as well as novel therapeutic targets are urgently required to reduce the rising EC-associated mortality and morbidity.

High TA has been reported in over 90% of all ECs [17]. hTERT and hTERC expression levels and TA measured by Telomere Repeat Amplification Protocol (TRAP) assay have been previously reported in the healthy endometrium [13] and in ECs [17,18]. However, dyskerin, which forms the foundation of the H/ACA lobe structure of the telomerase holo-enzyme, has not been studied in normal or pathological endometrium. Dyskerin protein is encoded by the *DKC1* gene located on the X chromosome [19] and it stabilises hTERC and enhances TA [20]. Dyskerin also has an extra-telomerase function in ribosomal biogenesis [21,22].

Available evidence suggests either the gain or loss of dyskerin to be carcinogenic [23,24]. High dyskerin levels have been reported in breast and prostate cancers [21,25,26] while decreased levels of dyskerin had been linked to carcinogenesis in the pituitary gland [27]. Low dyskerin levels observed in dyskeratosis congenita (DC) [28] have also been associated with an increased cancer-susceptibility before the age of 30 due to prematurely shortened telomers [29]. This observation is also in agreement with the only available animal model, where half of the hypomorphic *DKC1* mutant (*DKC1m*) mice (with decreased *DKC1* expression) developed various malignancies [22]. We, therefore, aimed to explore the role of dyskerin in endometrial carcinogenesis.

## 2. Results

### 2.1. In Silico Interrogation of the Cancer Genome Atlas (TCGA) Endometrioid and Serous EC Dataset Demonstrates Dysregulation of DKC1 to Be Associated with Poor Survival

**Figure 1:**
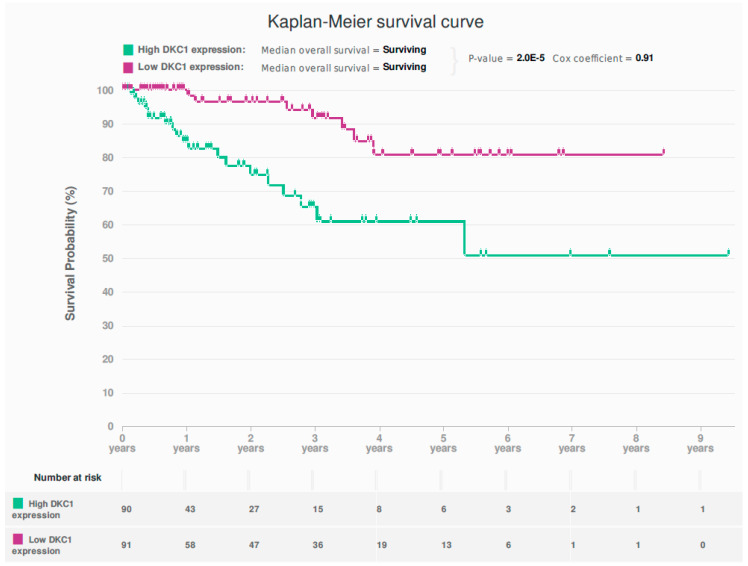
Kaplan-Meier survival curve for the association between DKC1 mRNA levels and overall survival (*p* = 2 × 10^−5^^,^ Cox-regression = 0.91) in The Cancer Genome Atlas (TCGA) dataset (endometrioid and serous endometrial cancer) {*n* = 477}.

Analysis of the TCGA dataset demonstrated a more than 2-fold upregulation of DKC1 RNA levels in 69/477 (14.65%) of the endometrioid and serous ECs compared with a set of normal endometrial samples obtained from 35 EC patients, at 2–3 cm distance from the cancer margin [30]. High *DKC1* expression was significantly associated with poor prognosis (*p* = 2 × 10^−5^, Cox-regression = 0.91) (Figure 1).

The mutation frequency of the *DKC1* gene in ECs was low (9/235, 3.69%) and consisted of mainly missense mutations that occurred without any *TERC* gene mutations (Figure S1).

Patients with ECs harboring a mutant *DKC1* gene seemed to have a better clinical outcome, compared with cancers carrying a wild-type *DKC1* gene (Figure S2). Twenty out of 464 (4.31%) ECs also demonstrated a copy number variation (mostly loss) of the *DKC1* gene. However, the TCGA dataset did not show a correlation of DKC1 RNA levels with the tumour grade (r^2^ = 0.19, *p* = 9.61 × 10^−23^) or clinical stage (r^2^ = 0.02, *p* = 7.27 × 10^−4^), (Figure S3A,B). Similarly, there was no correlation between RNA levels of *DKC1* with steroid receptor genes, *TERT* (r^2^ = 0.03, *p* = 3.43 × 10^−4^), (Figure S4A) or *TERC* (r^2^ = 0.04, *p* = 1.62 × 10^−4^), (Figure S4B). High DKC1 RNA levels were observed in *TP53* mutated ECs (*p* = 1.23 × 10^−8^) (Figure S5A) while in contrast, lower DKC1 RNA levels were observed in *FGFR2* (*p* = 7.90 × 10^−3^) (Figure S5B), *PTEN* (*p* = 2.90 × 10^−6^), *PIK3R1* (*p* = 0.02), (Figure S6A,B) and *CTNNB1* (*p* = 1.67 × 10^−3^) mutated ECs. No significant difference in *DKC1* RNA level was observed in *TERC*, *TERT*, *POLE*, *PIK3CA*, *KRAS*, and *ARID1A* mutated ECs compared with un-mutated EC samples.

### 2.2. Study Cohort

Patients’ demographic details are detailed in Table 1. Women with high-grade EC (HGEC) were significantly older than those with low-grade EC (LGEC) and healthy postmenopausal (PM) women (*p* < 0.001, *p* = 0.002, respectively). A significantly higher body mass index (BMI) was observed in the endometrial hyperplasia with a cytological atypia (EHA) group compared with the healthy PM women (*p* < 0.001) and in the EC group. There was an apparent trend for the LGEC group to have a higher BMI compared with the HGEC group (*p* = 0.06).

### 2.3. Dyskerin mRNA Was Lower in ECs Compared with Normal PM Endometrium

**Figure 2:**
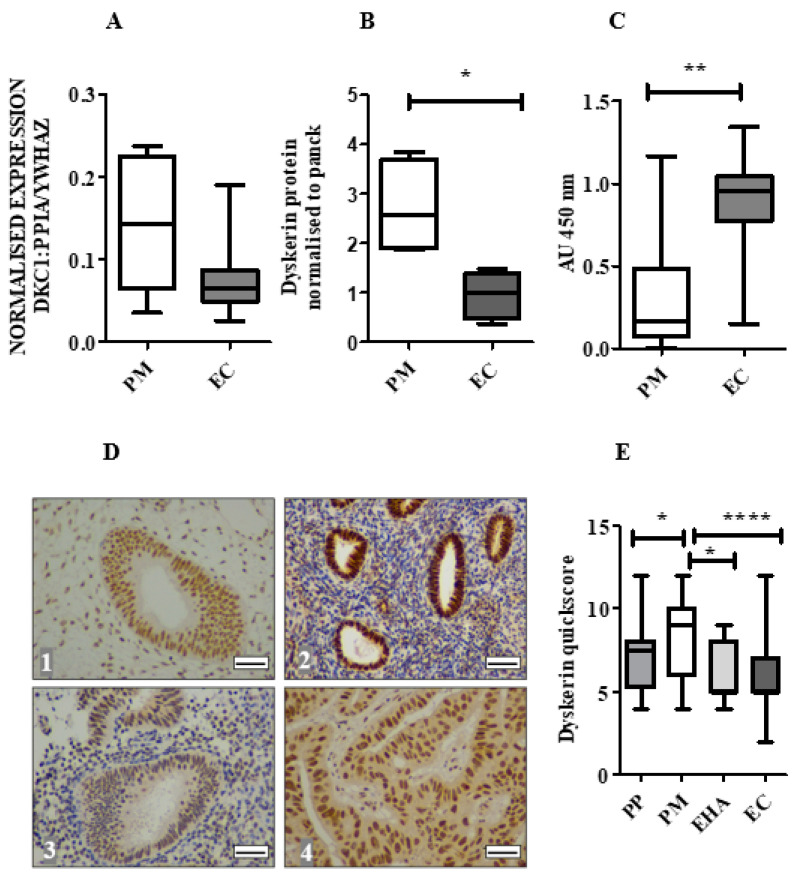
DKC1 mRNA and dyskerin protein in human endometrium. (**A**) DKC1 mRNA is normalised to geometric means of PPIA and YWHAZ and measured by qPCR in endometrial tissue samples: healthy postmenopausal (PM) (*n* = 6) and endometrial cancer (EC) (*n* = 22). Mann-Whitney test. (**B**) The amount of dyskerin protein was evaluated by immuno-blotting in healthy PM (*n* = 4) and EC (*n* = 4), Glyceraldehyde 3-Phosphate Dehydrogenase (GAPDH) was used to ensure equal loading of protein. Dyskerin protein levels in epithelial cells of tissue samples were analysed by normalising to pancytokeratin (panck). Mann-Whitney test, * *p* < 0.05. (**C**) Telomerase activity (TA) in healthy endometrial PM (*n* = 6) and EC (*n* = 32) was measured using a Telomere Repeat Amplification Protocol (TRAP) assay, Mann-Whitney test, ** *p* < 0.01. AU: arbitrary units (**D**) Representative microphotographs illustrating dyskerin IHC staining at the cellular level in endometrial samples in (**1**) normal proliferative phase (PP) endometrium, (**2**) healthy PM endometrium, (**3**) endometrial hyperplasia with cytological atypia (EHA) and (**4**) EC. Positive staining appears brown. Magnification 400×. Scale bar 50 μm. (**E**) Immunostaining quickscores for dyskerin protein in the human endometrium, healthy PP (*n* = 16), PM (*n* = 30), EHA (*n* = 15), EC (*n* = 109). Kruskal-Wallis test, * *p* < 0.05, **** *p* < 0.0001.

In contrast to the TCGA data in our patient samples, DKC1 mRNA levels showed a tendency towards downregulation in ECs in comparison with endometrium from healthy PM women (*p* = 0.06), (Figure 2A). No difference in the DKC1 mRNA level was observed between LGEC and HGEC.

### 2.4. Dyskerin Protein is Significantly Reduced in ECs When Compared with Healthy PM Control Endometrium

When EC samples were compared with healthy PM endometrium, immunoblotting demonstrated significantly reduced dyskerin protein levels (normalised to the epithelial marker pancytokeratin (*p* = 0.02, Figure 2B and Figure S7A), but significantly higher TA (*p* = 0.009, Figure 2C). IHC staining revealed the presence of dyskerin protein at a cellular level. In both epithelial and stromal cells of the healthy PP and PM endometrium, immunostaining was primarily localised in the nucleus and/or nucleolus (Figure 2D) and epithelial cells displayed stronger staining than the stroma. Dyskerin immunoscores were significantly lower in PP compared with PM (*p* = 0.03, Figure 2E). However, neither dyskerin quickscores nor DKC1 mRNA levels correlated with TA (Spearman r = −0.12, *p* = 0.16 and Spearman r = 0.04, *p* = 0.77, respectively).

### 2.5. Loss of Dyskerin Was a Feature of Precancerous and Cancerous Endometrial Epithelial Cells

**Figure 3:**
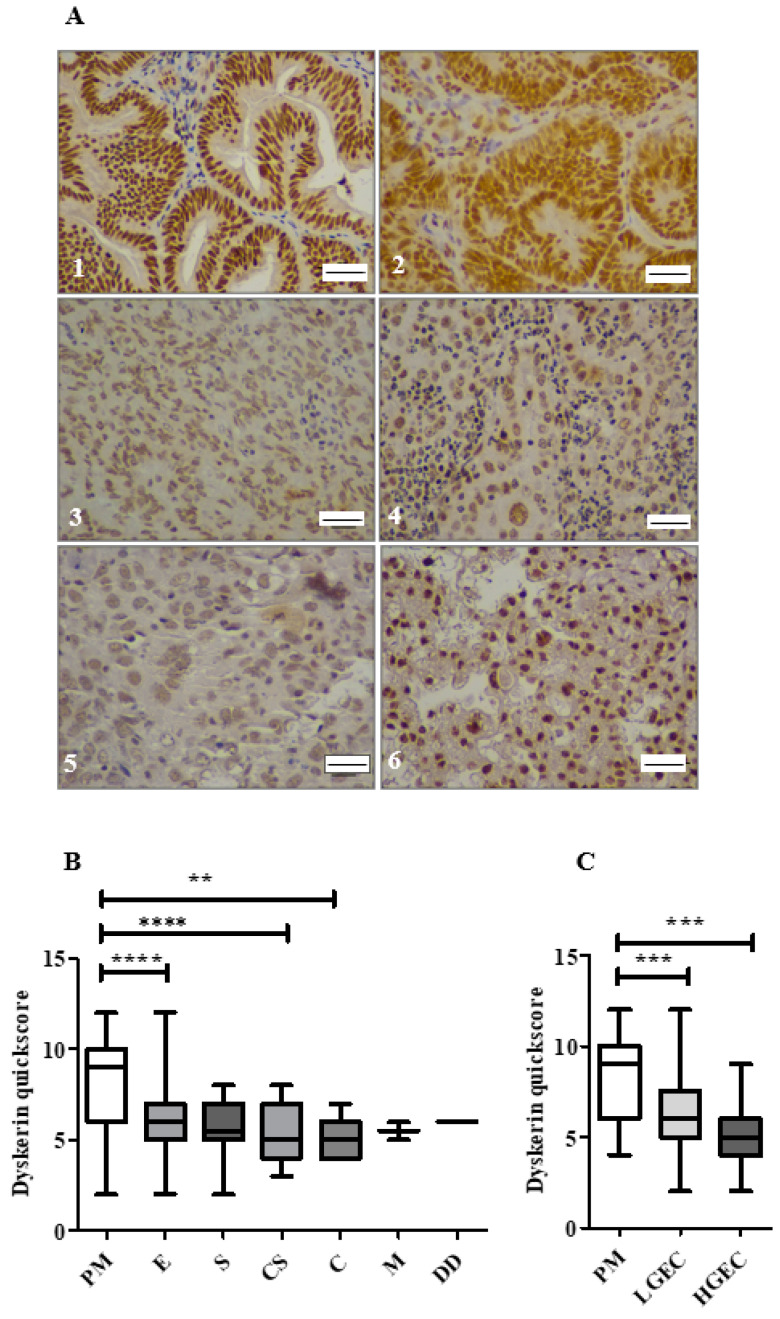
Immunostaining of dyskerin in endometrial cancer subtypes (*n* = 109). (**A**) Representative microphotographs of dyskerin in human ECs. (**1**–**3**) grade 1–3 endometrioid carcinoma, (**4**) serous subtype, (**5**) Carcinosarcoma and (**6**) clear cell carcinoma. Positive staining appears brown. Magnification 400×. Scale bar 50 μm. (**B**) Dyskerin immunoscores in healthy PM (*n* = 30) and various EC subtypes including endometrioid (E) (*n* = 65), Serous (S) (*n* = 12), carcinosarcoma (CS) (*n* = 19), clear cell carcinoma (**C**) (*n* = 10), mixed cell adenocarcinoma (M) (*n* = 2) and dedifferentiated EC (DD) (*n* = 1). ** *p* < 0.01, **** *p* < 0.0001. Kruskal-Wallis test. (**C**) Dyskerin immunoscores in human endometrial epithelium of healthy PM (*n* = 30), LGEC (*n* = 53) and HGEC (*n* = 56). *** *p* < 0.001. Kruskal-Wallis test.

**Figure 4:**
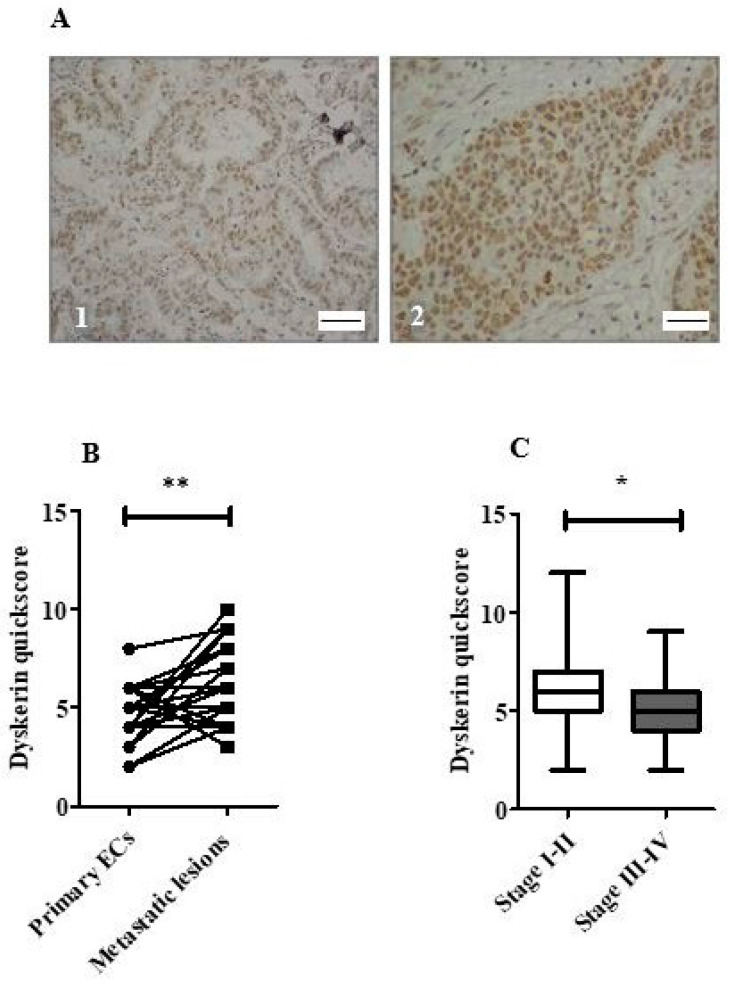
Dyskerin immunostaining in endometrial cancers. (**A**) Representative microphotographs illustrating dyskerin immunohistochemical staining in primary endometrial cancer (EC) (**1**) and matched metastatic lesion (**2**). Positive staining appears in brown. Magnification 400×, Scale bar 50 μm (**B**) Difference in dyskerin immunoscores in primary EC samples versus matched metastatic lesions (*n* = 30) each, ** *p* < 0.01. (**C**) Difference in dyskerin immunoscores between early-stage ECs (FIGO stage I–II) (*n* = 63) and advanced stage ECs (FIGO stage III–IV) (*n* = 43). Mann-Whitney test, * *p* < 0.05.

Dyskerin immunoscores were significantly lower in EHA and EC compared with normal PM endometrial epithelium (*p* = 0.01 and *p* < 0.0001, respectively, Figure 2E). All ECs in this cohort (Figure 3A) showed lower dyskerin scores compared with healthy PM endometrial tissue (Figure 3B), the difference was significant in endometrioid, carcinosarcoma and clear cell EC (*p* < 0.0001, *p* < 0.0001, and *p* = 0.002, respectively) and this reduction remained significant even when the histological LGEC (*p* < 0.001) and HGEC (*p* < 0.001) were considered separately (Figure 3C). There were no significant differences in dyskerin immunostaining among different EC subtypes or between LGEC and HGEC (Figure 3B,C). Metastatic lesions (Figure 4A) had significantly higher dyskerin immunoscores compared with their matched primary tumours (*p* = 0.003, Figure 4B), whereas ECs at advanced clinical stages (FIGO stages III&IV) had significantly lower dyskerin immunoscores compared with those at early stages (FIGO stages I&II, *p* = 0.04, Figure 4C).

### 2.6. Endometrial Epithelial Dyskerin Immunoscores Correlate with ERβ Scores and Inversely with the Ki67 Proliferation Index (PI)

Dyskerin immunoscores in endometrial samples correlated with ERβ immunoscores (Spearman *r* = 0.46, *p* < 0.0001), while an inverse correlation was found with the Ki67 PI (Spearman *r* = −0.34, *p* < 0.0001). No correlation was identified with other steroid receptors’ immunoscores (Table S1). Figure S7B shows immunostaining of dyskerin, Ki67 and steroid recepters. 

### 2.7. Survival Analysis

**Figure 5:**
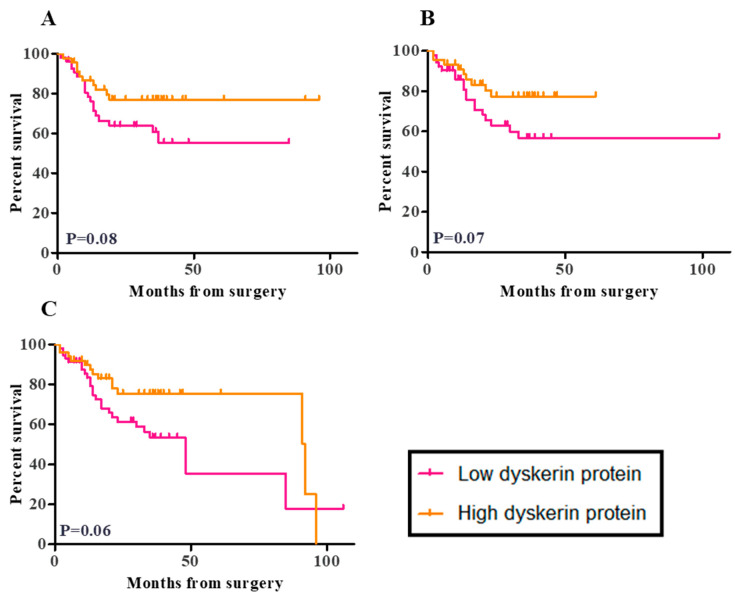
Kaplan Meier survival curves for the correlation between dyskerin immunoscores and patient outcome. (**A**) Disease-free survival (DFS), the median DFS time is undefined for low dyskerin and high dyskerin endometrial cancer groups. Hazard ratio (HR) = 1.92, 95% CI of the ratio (0.9200–4.006) (**B**) Cancer-specific survival (CSS), the median CSS time was undefined for low dyskerin and high dyskerin endometrial cancer groups. HR = 1.991, 95% CI of HR (0.9300–4.261) and (**C**) Overall survival (OS) in endometrial cancer samples (*n* = 109). Median OS time: Low dyskerin protein 8.00 months, High dyskerin protein 2.00 months. Low dyskerin/high dyskerin median survival Ratio: 0.5217, 95% CI of ratio (0.004444–1.039) HR = 1.841, 95% CI of HR (0.9667–3.506). A quickscore of 6 was chosen as the cut-off point. The *p* values relevant to the difference between low and high dyskerin protein levels in endometrial cancer groups that is visually represented in Kaplan Meier survival curves from the log-rank test.

According to the national guidance, patients were followed-up for at least 3 years after primary surgery in the two recruiting centers during the study period. By March 2020, follow-up data were available for 108 out of 109 women in our cohort [31]. During this follow-up period, there were 10 recurrent tumours and 38 deaths (27 as a result of disease progression and 11 from other causes). Worse outcomes were found in women with low dyskerin immunoscores. All outcomes analysed, including disease-free survival (DFS), cancer-specific survival (CSS), and overall survival (OS) suggested high dyskerin immunoscores to be potentially favourable (*p* = 0.08, *p* = 0.07, and *p* = 0.06, respectively, Figure 5A–C). For low dyskerin scores, the DFS hazard ratio (HR) = 1.92, 95% CI of HR (0.9200–4.006), CSS HR = 1.991, 95% CI of HR (0.9300–4.261), and OS HR = 1.841, 95% CI of HR (0.9667–3.506). When we only considered the endometrioid and serous ECs (similar to the selected TCGA EC dataset), low dyskerin immunoscores were still possibly suggestive of worse clinical outcomes with HR = 2.169, 95% CI of HR (0.7999–5.882), HR = 1.762, 95% CI of HR (0.5607–5.539) and HR = 1.698, 95% CI of HR (0.6925–4.165) for DFS, CSS, and OS, (Figure S7C–E), respectively. However, the *p* values were not significant (DFS, CSS, and OS; *p* = 0.1, *p* = 0.3, and *p* = 0.2, respectively) and confidence intervals were wide. These findings therefore need to be interpreted with caution and require future validation.

When clinicopathological features were considered, dyskerin immunoscores inversely correlated with cervical invasion (*p* = 0.01, Table S2).

### 2.8. In Vitro Transient Transfection of ISK Cells with the DKC1 Gene Resulted in Successful Overexpression of Dyskerin Protein

**Figure 6:**
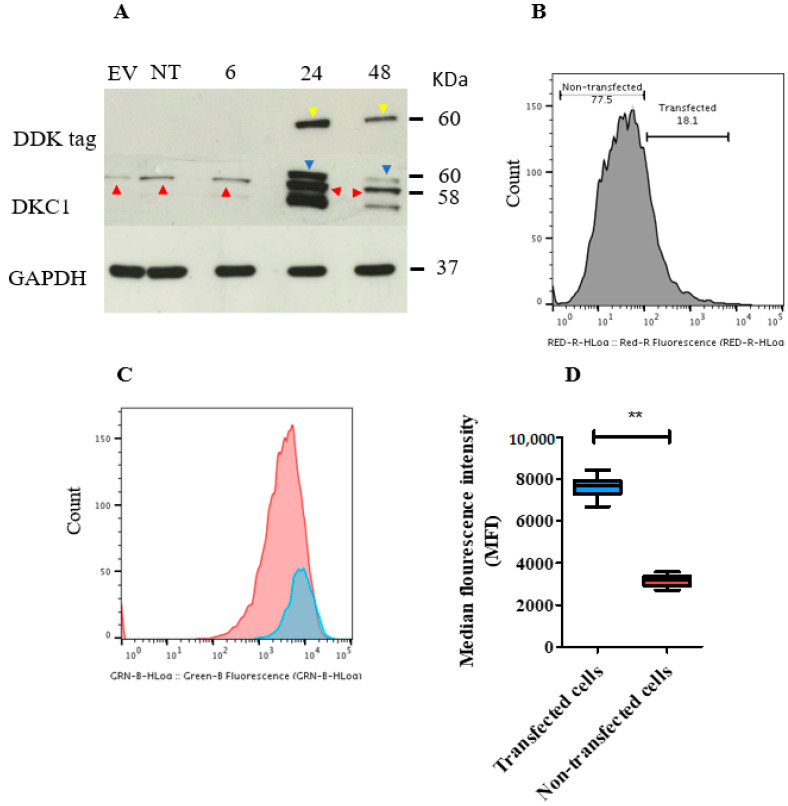
Transient overexpression of DKC1 in ISK cells. The plasmid and the empty vector (EV) used were tagged with the synthetic DYKDDDDK (DDK) protein to discern the transfected cells by using an anti-DDK antibody. (**A**) Immunoblot showing the level of dyskerin protein in DKC1 and EV transfected and non-transfected (NT) ISK cells. Cells were harvested 6, 24, and 48 h following transfection. Endogenous and exogenous dyskerin bands were present at the molecular weight of 58 and 60 KDa (red and blue arrows, respectively). DDK bands (yellow arrows) were observed at 60 KDa. Glyceraldehyde 3-Phosphate Dehydrogenase (GAPDH) bands were at 37 KDa. (**B**) Flow cytometric histogram showing the level of DDK tag protein in ISK cells. Cells positively stained with anti-DDK tag antibody represent transfected cells. (**C**) Cell proliferation was analysed using flow cytometry. ISK cells were stained with CellTrace Carboxyfluorescein Diacetate Succinimidyl Ester (CFSE) and fluorochrome-conjugated DDK Tag Antibody. Transfected cells (blue curve) and non-transfected cells (red curve). Higher proliferation is suggested when the curve was shifted to the left. (**D**) The difference in median fluorescence index (MFI) between transfected (T) and non-transfected ISK cells. ** *p* < 0.01, Wilcoxon signed-rank test.

A positive band corresponding to endogenous dyskerin was observed in negative controls (empty vector and non-transfected cells) and in transfected Ishikawa (ISK) cells at 6, 24, and 48 h after transfection (Figure 6A and Figure S8A). Exogenous dyskerin protein was first observed at 24 h and was still present at 48 h (although was decreased) in the *DKC1* transfected cells (Figure 6A and Figure S8B).

A signal at the correct molecular weight (MW) demonstrates that the DDK tag peptide was present in transfected cells at 24 and 48 h only (Figure 6A and Figure S8C). Immunofluorescent staining with an anti-dyskerin antibody demonstrated the presence of endogenous dyskerin, characterised by a punctate pattern that was exclusively localised in the nuclei of all cells (cells transfected with *DKC1* and empty vector and in non-transfected cells) (Figure S8D) Exogenous dyskerin was located both in the nucleus and in the cytoplasm and observed only in *DKC1* transfected cells (Figure S8D).

Flow cytometric analysis of ISK cells 48 h after transfection revealed the transfection efficiency to be 18.1% in the dyskerin transfected cells (Figure 6B), 11% in the empty vector-transfected cells (Figure S9A), and 1.76% in the non-transfected control (false positive level) (Figure S9B). Figure S10 shows different negative staining controls used in the transient transfection experiment and Figure S11 shows the empty vector control map.

### 2.9. Transient Overexpression of the DKC1 Gene Reduced ISK Cell Proliferation In Vitro

Overexpression of *DKC1* reduced cellular proliferation rates (Figure 6C), as demonstrated by a significantly higher median fluorescence intensity (MFI) of Carboxyfluorescein Diacetate Succinimidyl Ester (CFSE) staining in *DKC1* transfected cells compared with the non-transfected cells (*p* = 0.007) (Figure 6D). Dyskerin transfected cells also have a lower proliferation rate compared with those transfected with the empty vector using an immune-staining method (Figure S12).

## 3. Discussion

To our knowledge, this is the first study to examine the expression of the telomerase core-component dyskerin in human endometrium. It validates the findings of our in silico interrogation of a published, large TCGA *DKC1* gene alteration profile of endometrioid and serous ECs, using a cohort of human ECs containing all histological EC-subtypes with transcriptional and protein data. We have demonstrated that healthy PP and PM endometrium express the *DKC1* gene and have detectable dyskerin protein levels. Importantly, EC samples have significantly lower dyskerin protein levels when compared with healthy PM controls. Our findings are important for the following reasons: (i) we examined the endometrial dyskerin protein levels with immunoblotting and at the cellular level with IHC for the first time; (ii) our local patient cohort consisted of all EC subtypes, including carcinosarcoma, dedifferentiated, mixed-cell adenocarcinoma and clear cell cancer types, precancerous EH samples and metastatic EC lesions, as well as external control healthy endometrium (both healthy PM and PP samples) to increase the generalisability of the data; (iii) Importantly, our data suggests a possible better clinical outcome in ECs containing high levels of dyskerin protein in comparison with those with lower dyskerin levels. Our data, therefore, fill the gaps in the current literature, including the TCGA dataset.

Sufficient dyskerin levels are required for competent TA to overcome telomere attrition [28]. *DKC1* dysregulation is associated with a high incidence of cancers in DC patients (reduced *DKC1*) and in *DKC1* hypomorphic mice [22], but no reports are available of DC associated with EC. Although high TA in over 90% of ECs had been reported, that is usually associated with short telomeres [32].

Examination of the TCGA dataset only identified *DKC1* out of the three core telomerase components to have an altered gene expression, with a prognostic relevance in ECs. Our data also suggests that dyskerin protein levels in ECs correlate with differences in patient outcomes. Variable dyskerin levels are also reported in other cancers [25,33]. Data from our cohort and the TCGA dataset jointly suggests a dysregulation of dyskerin in ECs. However, our cohort results differ from the TCGA data, and this discrepancy may be due to different “normal controls” used in the two studies and the fact that we examined protein rather than only mRNA levels. It is important to appreciate that endometrioid/serous ECs included in the TCGA data usually originate from a background of EHA or endometrial intraepithelial neoplasia (EIN). Thus, the normal tissue within 2 cm from the tumour included in the TCGA data as normal endometrium is likely to include hyperplastic tissue or EIN lesions. Our cohort data is more generalisable since we histologically confirmed our external healthy control tissue obtained from a well-characterised and age-matched population.

Many studies on different cancer types reported a high expression of the *DKC1* gene and dyskerin protein to be associated with poor prognosis [25,34]. For example, in contrast to our results on ECs, reports on prostate, hepatocellular carcinoma, and colorectal cancers showed that high *DKC1* is commonly associated with an extensive tumour growth pattern [25,34,35]. Recently, Elsharawy et al. showed that high DKC1 mRNA or protein levels in breast cancer associated with poor patient outcome and unfavourable clinicopathological characteristics [26]. In a recent study in breast cancer, *DKC1* over-expression associated with unfavourable clinicopathological characteristics and poor outcome [26]. Two publicly available “Breast Cancer Gene-Expression Miner v4.3” [26] and TCGA breast cancer datasets revealed high DKC1 mRNA levels to significantly correlate with larger tumour size, higher tumour grades, and poor prognosis. At the protein level, high dyskerin protein levels, whether in the nucleus and/or nucleoli, were reported to be associated with aggressive features in breast cancer [26]. In those tissues, however, carcinogenesis is associated with reactivation of TA compared with healthy tissues [36], whereas high TA is a feature of healthy PP endometrium [37]. Therefore, we suggest that ECs are different in this respect, and consequently, endometrial carcinogenesis seems to be associated with a reduction of dyskerin protein and *DKC1* gene expression.

Advanced primary ECs (stage-III and IV) had significantly lower dyskerin protein levels compared with early stages, suggesting that dyskerin protein may be useful in stratifying EC patients for further therapy after primary surgery. Prior reports have suggested metastatic EC lesions to demonstrate a regressed phenotype when compared with the matched primary tumour [38] which agrees well with our dyskerin data. The observed dyskerin loss we report may also produce a pro-oxidant environment in EC cells as demonstrated in other cancer cells [39]. Therefore, reduced dyskerin protein in the context of the excessive cellular division in ECs may contribute to genomic instability that is known to be present, particularly in more advanced ECs. Dyskerin deficiency may also contribute to carcinogenesis by adversely influencing the translational machinery via affecting the balance in ribosomal proteins [33] and by modifying the splicing of specific pre-mRNAs, or by altering the level of certain snoRNAs [40,41]. These mechanistic aspects need to be examined in future studies.

The healthy quiescent PM endometrium with absent cellular proliferative activity had high dyskerin levels. TA positively correlated with endometrial epithelial proliferation [13] and the downregulation of dyskerin protein in ECs in comparison with the healthy PM endometrium we observe, occurred in a background of high TA and Ki67 levels [18]. This suggests a tumour suppressor function [22] and an inhibitory effect on endometrial epithelial cell proliferation for dyskerin in ECs. Therefore, we sought to examine the functional consequence of overexpressing the *DKC1* gene on cell proliferation using a cell line that reflecting low grade ECs. Dyskerin knock-out is lethal, and thus all cells (independent of detectable TA) express the dyskerin gene/protein. The available *in vitro DKC1* gene manipulation studies had only examined knocking down of the *DKC1* gene [25] but not over-expression and they also did not examine cellular proliferation as an outcome. Knock-down studies in prostate carcinoma cells demonstrated dyskerin to be crucial in protein biosynthesis [25]. Both high and low dyskerin is associated with carcinogenesis [25], which fundamentally demonstrates the cardinal feature of excessive cellular proliferation. Our data demonstrates a consequential reduction in cell proliferation when dyskerin is overexpressed in the EC cell line, therefore establishing a functional effect of dyskerin on cell proliferation for the first time.

Reduction in dyskerin rendered human breast cancer cells to be more prone to incorrect codon recognition and induced a defect in rRNA uridine modification resulting in altered ribosome activity [42]. Low dyskerin expression levels correlated with poor overall survival of Chronic Lymphocytic Leukaemia (CLL) patients following chemotherapy [33]. The authors proposed that reduced dyskerin may cause a reduction of the synthesis of subsets of ribosomal proteins, and selectively alters the translatome of the cancer cells to increase their aggressiveness [33]. Loss of dyskerin dysregulates initiation of translation of tumour suppressor proteins such as p53 and p27 and thus may promote carcinogenesis [27,43]. In addition, dysregulation of p53 translation has been reported in DC patients with reduced dyskerin function via its internal ribosome entry segment being impaired resulting in increased cellular proliferation [44,45]. The exact mechanistic pathway by which dyskerin exerts this observed anti-proliferative effect on EC cells remains to be explored in future studies.

We used opportunistic recruitment and available archived samples in our study to answer our research question. This meant inclusion of retrospectively collected patient samples, and only a small proportion of cases seen in the centres over that time period were included in the study. Although this is a limitation of our study, since no previous data available for the levels of dyskerin protein in the EC, our study, which included a relatively sizeable EC cohort with associated important clinical details, fills the current gap in the literature, and provides significantly different results to inform sample sizes for adequately powered studies in the future.

Another limitation to our study is that we have included a similar number of LGEC and HGEC, meaning the stage distribution was skewed towards metastatic disease in the local cohort. This caused our sample to be deviated from the real incidence of non-endometrioid EC; however, this offers us a better assessment of HGECs, which are usually associated with poor prognosis. Although we have recruited women without known endometrial pathology as normal controls for EC samples, a potential limitation would be that all these control women were undergoing hysterectomy for a non-cancerous pathology, thus they may not represent asymptomatic and completely healthy normal women. Therefore, our findings require further validation in future prospective studies.

Endometrial TA and hTERT levels have been shown to be under hormonal regulation [13] and correspondingly, endometrial dyskerin immunoscores revealed a significant positive correlation with ERβ immunostaining. This may suggest dyskerin expression to be under estrogen regulation mainly via ERβ. ERβ is known to harness the estrogen-driven mitotic effect of ERα [46], therefore inducing dyskerin levels may also be a part of the ERβ-associated inhibition of the endometrial epithelial proliferation. Further studies are required to examine the hormonal regulation of dyskerin in human endometrium.

## 4. Materials and Methods

### 4.1. Study Groups:

#### 4.1.1. TCGA Database Cohort

The publicly-available TCGA cohort of uterine cancers included data for RNA levels (*n* = 477), copy number variation (*n* = 464), and somatic mutation (*n* = 235); for *DKC1*, the data were interrogated using Illumina’s Base Space Cohort Analyzer application (BSCA) [47] (Software; https://www.illumina.com/informatics/research/biological-data-interpretation/nextbio.html; Illumina, San Diego, CA, USA) [48]. The normal endometrial controls were obtained from 35 EC patients at 2–3 cm distance from the cancer margin [30].

#### 4.1.2. Local Study Cohort

The study was performed in accordance with the Declaration of Helsinki. The Liverpool and Cambridge Adult Research Ethics Committees (LREC 09/H1005/55, 11/H1005/4 and CREC 10/H0308/75) approved the study. A total of 175 endometrial samples collected from women undergoing hysterectomy in the Liverpool Women’s Hospital (LWH) and Lancashire Teaching Hospitals Trusts from 2009 to 2017 were included. Our cohort included a total of 15 endometrial samples with histological hyperplasia and cytological atypia were collected from patients undergoing hysterectomy at LWH. Out of these, three women had prior histological evidence of hyperplasia in an endometrial biopsy with ongoing symptoms of irregular or heavy menstrual bleeding; another 12 samples were from paraffin blocks of hyperplastic changes adjacent to EC that were retrieved from the Histopathology Department archive at the Royal Liverpool University Hospital.

Additionally, a total of 109 histologically confirmed EC samples from patients who underwent staging operations at LWH or at Lancashire Teaching Hospitals during the period between 2009 and 2017 were also recruited to the current study. Out of those 109 samples, 60 were pipelle biopsies collected at the time of their hysterectomy as part of their primary surgical treatment for EC. The remaining samples were paraffin blocks retrieved from the Histopathology Department archives at the Royal Liverpool University Hospital, or Lancaster Teaching Hospital. Paraffin blocks of 30 metastatic lesions from some of these women with ECs that were obtained during the same primary surgery were also studied. The sites of metastases were as follows: lymph nodes (*n* = 11), omentum (*n* = 7), parametrium (*n* = 5), soft tissue (*n* = 4), fallopian tube (*n* = 1), cervix (*n* = 1), and urinary bladder (*n* = 1).

None of the included EHA or EC patients had received hormonal treatment, chemotherapy, or pelvic radiation prior to surgery when the endometrial samples were harvested.

Demographic data are shown in Table 1. Experienced gynaecological pathologists confirmed the histological type and grade of EC specimens according to FIGO classification [49]. Considering the clinical relevant outcome, we further categorised the EC samples as low-grade (LGEC), consisting of grade 1 and grade 2 endometrioid EC or high-grade (HGEC), including grade 3 endometrioid, serous, clear cell carcinomas, carcinosarcoma, Mixed cell adenocarcinoma, and dedifferentiated ECs [43,50] as shown in Table 1. Healthy endometrial tissue specimens were collected from women undergoing hysterectomy for benign gynaecological pathologies such as prolapse or heavy bleeding without a known endometrial pathology (a full-thickness samples). Since EC is a disease mainly affecting postmenopausal (PM) women, 35 age-matched healthy endometrial tissue samples were included as an external control group. Some previous authors have suggested that the proliferative phase (PP) control samples were more suitable as a healthy comparator because EC is a proliferative disease; therefore, we also included a second external control group of 16 normal healthy premenopausal endometrial PP samples. Samples from healthy women were thus assigned to premenopausal (PP) and postmenopausal (PM) groups according to the last menstrual date and histological criteria [51].

### 4.2. Collection of Endometrial Samples

Once the uterus was removed at hysterectomy, in theatre, endometrial biopsies were collected by a trained member of the research team or the operating surgeon. Full-thickness endometrial biopsies were obtained from healthy women undergoing a hysterectomy, as previously described by cutting a thin slice of endometrium attached to underlying myometrium after opening the anterior uterine aspect in the coronal plane [52]. In order to avoid interference with pathological diagnosis and staging, samples from women undergoing primary surgery for EC were collected by using a pipelle suction curette (Laboratoire C.C.D., Paris, France). Each sample was split into two to three containers: (i) 15 mL 10% neutral buffered formalin (10% NBF) (Sigma, Dorset, UK) for immunohistochemistry study; (ii) 0.5 mL RNAlater (Sigma, Dorset, UK) for RNA extraction and PCR analysis; (iii) Immediately snap-frozen for immunoblotting and TRAP analysis.

### 4.3. Immunohistochemistry (IHC)

IHC was performed on 3 μm serial sections of formalin-fixed, paraffin-embedded endometrial tissue employing heat-induced antigen retrieval, and the ImmPRESS Polymerized Reporter Enzyme Staining System (Vector Laboratories, Peterborough, UK) as previously described [38]. The primary antibody sources, concentrations, and incubation conditions are detailed in Table S3.

Immunoreactivity for nuclear dyskerin was assessed using a modified quick score as previously described [53]. The four steroid receptors were evaluated semi-quantitatively using a four-tiered Liverpool endometrial steroid quick score (LESQS) as previously described [38]; the Ki67 proliferative index (PI) was evaluated as the percentage of positive cells of any intensity [38].

### 4.4. Real-Time qPCR

RNA was extracted, quantified, and reverse transcribed as previously described [53]. cDNA was amplified using iTaq universal SYBR Green supermix and CFX Connect Real-Time System (Bio-Rad, Hertfordshire, UK). Primers and reaction conditions are listed in Table S4 [35,54,55]. The 2^−ΔΔCt^ method was used to calculate relative transcript level. *DKC1* expression was normalised to *YWHAZ* and *PPIA* reference genes [56,57].

### 4.5. TRAP Assay

TA was measured using a TeloTAGGG™ TRAP assay (Sigma-Aldrich, Dorset, UK) according to the manufacturers’ manual and as previously described [13]. Absorbance was measured at 450 nm in an Omega spectrophotometer (BMG, Labtech, UK) and presented as arbitrary units (AU). A total of 1 μg of protein was used per sample, and negative controls without protein were included and their absorption was subtracted from those of the samples.

### 4.6. Cell Culture

Cultured ISK cells were maintained in Dulbecco modified Eagle medium/F12 (DMEM/F12) supplemented with 10% (*v*/*v*) fetal bovine serum (FBS), L-glutamine, and penicillin/streptomycin at 37 °C in a 5% CO_2_ atmosphere. All cell culture reagents were purchased from Sigma-Aldrich (Dorset, UK) as previously described [58].

### 4.7. Transient Transfection

Transfection of ISK cells was performed twenty-four hours after seeding cells on 6 well plates at a density of 0.5 × 10^6^ cells/well by using a mixture of MYC-DDK tagged Dyskerin plasmid (OriGene Technologies, Rockville, MD, USA, 3 μL) with Lipofectamine 2000 (Thermo Fisher Scientific, Loughborough, UK, 9 μL). The plasmid or the Lipofectamine was diluted in 250 μL of Gibco Opti-MEM I (Thermo Fischer Scientific, Loughborough, UK). Empty vector (Myc-DDK tagged pCMV6-Entry) (OriGene Technologies, Rockville, MD, USA) and non-transfected cells were used as negative controls. The diluted plasmids and Lipofectamine were incubated for 20 min at room temperature. In the meantime, DMEM/F12 culture medium with supplements (FBS, l-glutamine and antibiotics) was replaced with the same medium but without antibiotics. A total of 4–6 h after transfection, the medium containing transfection reagents was removed and replaced with a fresh one supplemented with FBS, L-glutamine, and antibiotics. The cells were incubated at 37 °C, 5% CO_2_. The plasmids used were tagged with the synthetic DYKDDDDK Tag (DDK) Tag protein to discern the transfected cells by using an anti-DDK antibody.

### 4.8. SDS-PAGE and Immunoblotting

Protein lysates from homogenised tissues and cultured cells were extracted using a Radioimmunoprecipitation assay (RIPA) buffer (Sigma-Aldrich, Dorset, UK) supplemented with protease inhibitor (Sigma-Aldrich, Dorset, UK) and phosphatase inhibitor (PhosSTOP, Roche Diagnostics Ltd., Burgess Hill, UK). Lysates were analysed by SDS-PAGE under reducing conditions on precast 12% gels (Mini-PROTEAN TGX, Bio-Rad, Hertfordshire, UK) and transferred to an Immune-Blot polyvinylidene difluoride (PVDF) membrane (Bio-Rad, Hertfordshire, UK). The primary antibody sources, concentrations, and incubation conditions are detailed in Table S3. Horseradish peroxidase (HRP)-linked secondary antibodies were from Thermo Fisher Scientific (Loughborough, UK). Signal detection was performed using SuperSignal West Dura Extended Duration chemiluminescent Substrate (Thermo Fisher Scientific, Loughborough, UK) and CL-Xposure film (Thermo Fisher Scientific, Loughborough, UK).

### 4.9. Immunofluorescence

In order to differentiate between endogenous dyskerin and exogenous overexpressed protein, immunofluorescent staining of dyskerin was performed, allowing examination of their respective location within ISK cell. Rabbit anti-dyskerin antibody (Santa Cruz Biotechnology, Dallas, TX, USA, 1:200) was added to the fixed cells, which were seeded onto coverslips in a 6 well plate. The secondary antibody was Alexa Fluor Anti-rabbit IgG (H + L), (Alexa Fluor 488 Conjugate), (Cell Signalling Technology, London, UK, 1:1000). The cells were mounted in DAPI containing medium (Vector Laboratories, Peterborough, UK,). Fluorescence was visualised with a Nikon Eclipse 50i microscope using NIS elements F software (Nikon, Tokyo, Japan). Rabbit and mouse isotype control antibodies were used as negative controls. Antibody details are provided in Table S3.

### 4.10. CFSE Labelling and Flow Cytometry

ISK Cells were initially labelled with CellTrace CFSE (Thermo Fisher Scientific, Loughborough, UK) according to manufacturers’ guidelines, then fixed, permeabilised, and labelled with fluorochrome-conjugated primary antibody (anti-DYKDDDDK (DDK) Tag antibody [iFluor 647], Genscript, Piscataway, NJ, USA) and the corresponding fluorochrome-conjugated isotype control antibody (Alexa Fluor 647 antibody, Biolegend, UK). The cells were then incubated (1 h at 37 °C in the dark). A Guava EasyCyte flow cytometer (Millipore, Watford, UK) was used to perform flow cytometry and FlowJo v10 (Becton Dickinson, Franklin Lakes, NJ, USA) was used for data analysis.

### 4.11. Statistical Analysis

Statistical differences between groups were calculated by non-parametric tests (Kruskal–Wallis or Mann-Whitney U-test) using the Statistical Package for the Social Sciences (SPSS) version 24 (IBM Corp, Armonk, NY, USA). Descriptive values were presented as median and range. Graphs were plotted using GraphPad prism 5 (GraphPad Software, San Diego, CA, USA). The correlation between immunostaining scores was determined with a Spearman test and the association between dyskerin immunoscores and the multiple clinicopathological parameters were evaluated by Pearson’s Chi-square test. The duration of DFS was measured from the date of surgery to the date of EC recurrence or death from EC, while the CSS duration was calculated from the date of surgery to the date of death from EC. OS duration was measured from the date of surgery to the date of death caused by any reason. All the observations were censored at the last date at which the patient was seen. Kaplan-Meier survival curves were constructed. Cumulative proportions of survivors in the high and low level of dyskerin protein were compared using Log-rank test. A significant difference between groups was only achieved with *p* value < 0.05. Significance values have been adjusted by Bonferroni correction for multiple tests.

## 5. Conclusions

Taking these observations together, we concluded that dyskerin protein and the *DKC1* gene are expressed in healthy endometrium [59] and in ECs. Low dyskerin immunoscores were potentially associated with worse outcomes, suggesting a possible prognostic relevance. Furthermore, increased dyskerin protein levels in ISK cells seem to inhibit cell proliferation, and therefore, the observed loss of dyskerin in endometrial cancer tissue may contribute to the increased cell proliferation and the progression of these ECs.

The detailed role of dyskerin in normal endometrial regeneration as well as in pathological conditions such as EC in the context of telomerase biology is yet to be determined. Since TA is known to play an intricate role in endometrial epithelial cellular proliferation, further studies elucidating the associated telomerase and other functions of dyskerin in the human endometrium and in EC are warranted.

## Figures and Tables

**Table 1:** Demographic features of study groups.

Study Groups	No	%	* Age (Years)	** BMI (kg /m^2^)
1. Healthy (total)	51			
• *Proliferative phase*	16		40 (30–57)	27 (18–41)
• *Postmenopausal*	35		63 (40–85)	26 (20–40)
2. Endometrial hyperplasia	15		55 (48–72)	36 (24–57)
3. Endometrial cancer (total)	109		68 (37–96)	30 (20–54)
LGEC	53	48.6	64 (37–89)	32 (21–54)
• *Endometrioid Grade 1*	34	31.2	64 (46–89)	33 (21–53)
• *Endometrioid Grade 2*	19	17.4	60 (37–78)	30 (22–54)
HGEC	56	51.4	73 (48–96)	30 (20–43)
• *Endometrioid Grade 3*	12	11	68 (54–96)	28 (24–43)
• *Serous*	12	11	76 (64–87)	29 (23–39)
• *Clear cell*	10	9.1	74 (48–82)	30 (27–39)
• *Carcinosarcoma*	19	17.4	78 (60–89)	26 (20–37)
• *Dedifferentiated*	1	0.9	79	32
• *Mixed cell adenocarcinoma*	2	1.8	63 & 66	
• *Metastatic EC*	34		68 (27–96)	28 (21–43)

Abbreviations: Body mass index (BMI); high-grade endometrial carcinoma (HGEC); low-grade endometrial cancer (LGEC); * Data expressed as median (range). ** BMI data were available for only 161 cases.

## Data Availability

Data is contained within the article or supplementary material.

## Supplementary Materials

The following are available online at https://www.mdpi.com/2072-6694/13/2/273/s1, Table S1: Correlation between dyskerin quick scores with steroid receptors immunoscores and Ki67 proliferative index (PI) in endometrial samples. Table S2: Association between dyskerin protein immunoscores and clinicopathological parameters in EC samples. Table S3: Primary antibodies and conditions for IHC, immuno blotting and immunofluorescence. Table S4: Primer sequences used for qPCR amplification. Figure S1: Multi co-occurrence plot of somatic mutation, RNA level and copy number variation of *DKC1, TERT, and TERC* genes. Figure S2: Kaplan-Meier survival curve for the association between mutation status of the DKC1 gene and overall survival in endometrial cancers (ECs). Figure S3: Violin plot demonstrating the correlation between DKC1 RNA levels with tumour grades and stages. Figure S4: DKC1 RNA levels correlation with TERT and TERC RNA levels in The Cancer Genome Atlas (TCGA) dataset (endometrioid and serous endometrial cancers). Figure S5: Violin plot showing the association between DKC1 RNA levels with the mutation status: normal, mutant, or wild type + silence of TP53 and FGFR2 genes. Figure S6: Violin plot showing the association between DKC1 RNA levels and mutation status: normal, mutant or wildtype + silenceof PTEN and PIK3R1genes. Figure S7: Whole immunoblots, dyskerin, Ki67, and steroid receptors’ immunohistochemical staining and the association of dyskerin immunoscores with survival outcome in endometrioid and serous EC samples {n = 77}. Figure S8: Transient overexpression of *DKC1* gene in ISK (Ishikawa) cells (Immunoblotting and immunofluorescence experiments). Figure S9: Negative controls used in the transient transfection experiment. Figure S10: Negative staining controls used in transient transfection experiment. Figure S11: pCMV6-Entry vector map. Figure S12: Transient overexpression of DKC1 in ISK cells.
