# Supplementary material for: Aberrant Dyskerin Expression Is Related to Proliferation and Poor Survival in Endometrial Cancer
Source: Cancers (Basel). 2021 Jan 13;13(2):273. doi: 10.3390/cancers13020273 (PMC7828388; doi:10.3390/cancers13020273)
Supplement: Supplementary file 1 [file cancers-13-00273-s001.pdf]

cancers

MDPI

Supplementary Materials: Aberrant Dyskerin Expression is Related to Proliferation and Poor Survival in Endometrial Cancer

Rafah Alnafakh, Gabriele Saretzki, Angela Midgley, James Flynn, Areege M. Kamal, Lucy Dobson, Purushothaman Natarajan, Helen Stringfellow, Pierre Martin-Hirsch, Shandya B. DeCruze, Sarah E. Coupland and Dharani K. Hapangama

Citation: Alnafakh, R.; Saretzki, G.; Midgley, A.; Flynn, J.; Kamal, A.M.; Dobson, L.; Natarajan, P.; Stringfellow, H.; Martin-Hirsch, P.; DeCruze, S.B.; et al. Aberrant Dyskerin Expression is Related to Proliferation and Poor Survival in Endometrial Cancer. *Cancers* **2021**, *13*, x. https://doi.org/10.3390/xxxxx

Received: 1 November 2020
Accepted: 8 January 2021
Published: date

Publisher's Note: MDPI stays neutral with regard to jurisdictional claims in published maps and institutional affiliations.

Copyright: © 2021 by the authors. Licensee MDPI, Basel, Switzerland. This article is an open access article distributed under the terms and conditions of the Creative Commons Attribution (CC BY) license (http://creativecommons.org/licenses/by/4.0/).

Table S1. Correlation between dyskerin quick scores with steroid receptors immune scores and Ki67 proliferative index (PI) in endometrial samples.

	Test	Ki67	ERα	ERβ	PR	AR
Dyskerin	Spearman r	−0.34	0.21	0.46	0.27	0.17
	* p value	<0.0001	0.01	<0.0001	0.001	0.04

* $p < 0.05$.

Table S2. Association between dyskerin protein immune scores and clinico-pathological parameter in EC samples. Quick score of 6 was chosen as the cut-off point.

			Dyskerin				
**Variables**		**Total**	**<6**	**%**	**≥6**	**%**	*** p Value**
Age	<65	105	23	53	20	47	0.49
	≥65		29	47	33	53	
BMI	<30	85	16	41	23	59	0.53
	≥30		22	48	24	52	
grade	Low Grade	109	22	42	31	58	0.04
	High Grade		34	61	22	39	
FIGO stage	I-II	106	30	48	33	52	0.4
	III-IV		24	56	19	44	
Myometrial invasion	<50%	109	27	48	29	52	0.49
	≥50%		29	55	24	45	
LVSI	No	108	27	45	33	55	0.16
	Yes		28	58	20	42	
Cervical invasion	No	107	31	42	42	58	0.01
	Yes		23	68	11	32	
Extrauterine invasion	No	109	32	46	37	54	0.17
	Yes		24	60	16	40	

Abbreviation: Lymphovascular space invasion: LVSI. * $p < 0.05$.

Table S3. Primary antibodies and conditions for IHC, immunoblotting and immunofluorescence.

Primary Antibody	Type	Clone	Supplier		Dilution	Incubation Condition	
						Time (hour)	Temp (°C)
IHC				HIAR * (min)			
Dyskerin	P		Santa Cruz biotechnology [1]	3	1:500	20	4
ERα	M	6F11	NovoCastra [2]	2	1:50	2	18
ERβ	M	PPG5/10	Abcam [3]	2	1:50	20	4
PR	M	PgR 636	DAKO [4]	2	1:1000	1	18
AR	M	AR441	DAKO [4]	2	1:75	20	4
Ki67	M	MM1	NovoCastra [2]	2	1:200	20	4
Immunoblotting							
Dyskerin	P		Santa Cruz biotechnology [1]		1:1000	20	4
Pancytokeratin (panck)	M	C-11+PCK-26+CY-90+KS-1A3+M20+A53-B/A2	Sigma-Aldrich [5]		1:8000	20	4
DYKDDDDK Tag Antibody	M	5A8E5	Genscript [6]		1:1000	20	4
GAPDH	P		Sigma-Aldrich [5]		1:10000	20	4
Immunofluorescence							
Dyskerin	P		Santa Cruz biotechnology[1]		1:200	1	4

Abbreviations: HIAR: Heat-induced antigen retrieval; P: Polyclonal; M: Monoclonal. * Heat induced antigen retrieval by pressure cooking in citrate buffer pH 6. [1] Dallas, Texas, USA; [2] Newcastle, UK; [3] Cambridge, UK; [4] Ely, Cambridgeshire, UK; [5] Dorset, UK.; [6] Genscript, NJ, USA.

Table S4. Primer sequences used for qPCR amplification.

Primer	Sequence	Amplicon	Efficiency (%)	Reference
DKC1	F:5′-CTCGGAAGTGGGGTTTAGGT-3<br>R:5′-ACCACTTCAGCAACCACCTC-3	166	98%	35
PPIA	F:5′- AGACAAGGTCCCAAAGAC-3<br>R:5′- ACCACCCTGACACATAAA-3	118	100.10%	54
YWHAZ	F:5′-CGTTACTTGGCTGAGGTTGCC-3<br>R:5′GTATGCTTGTT-GTGACTGATCGAC-3	69	94.30%	55

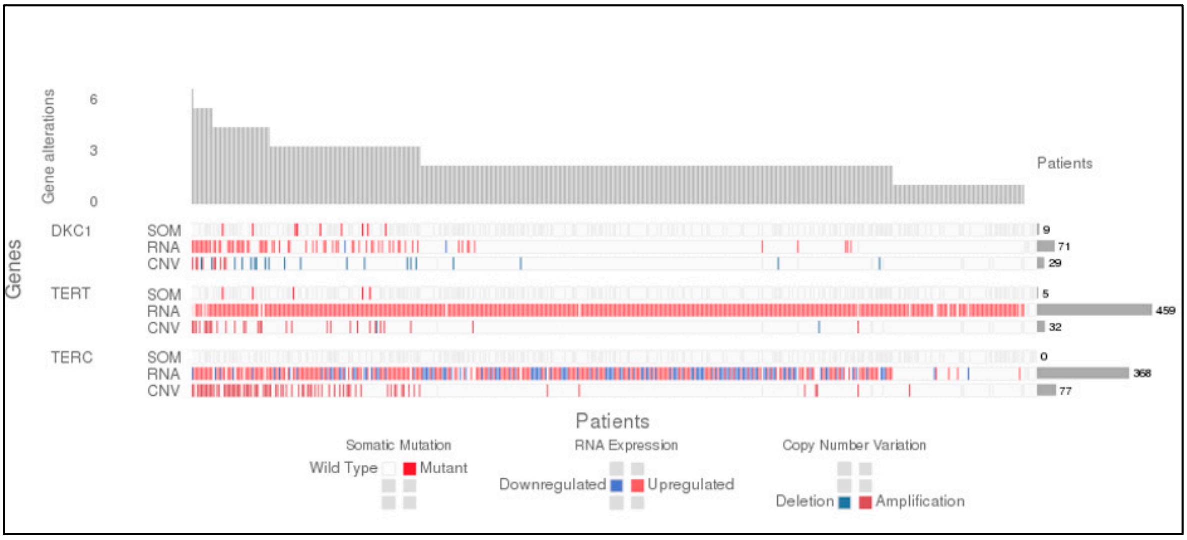

Figure S1. Multi co-occurrence plot of somatic mutation, RNA level and copy number variation of: *DKC1, TERT* and *TERC* genes in The Cancer Genome Atlas (TCGA) dataset (endometrioid and serous endometrial cancer) (*n* = 477).

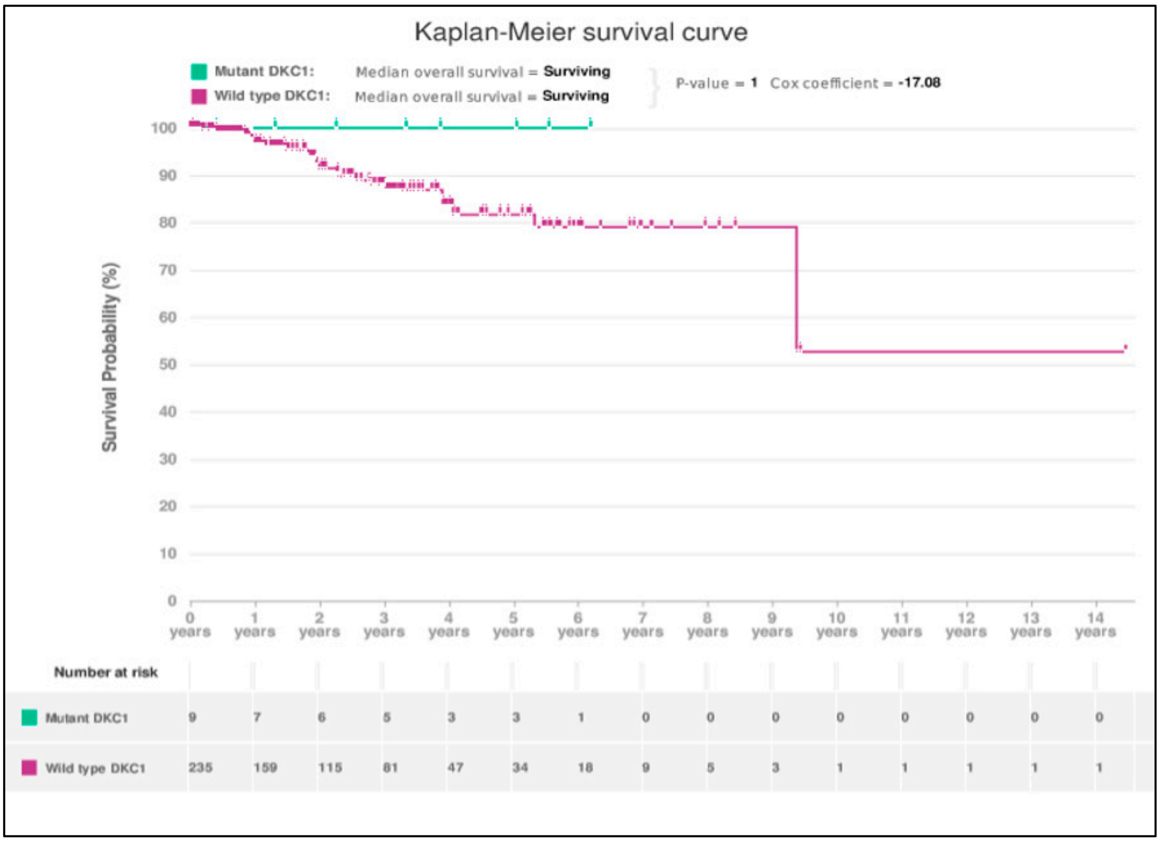

Figure S2. Kaplan-Meier survival curve for the association of mutation status of the *DKC1* gene and overall survival in endometrial cancers (ECs). ECs harbouring a mutant DKC1 gene (green) and ECs carrying the wildtype DKC1 gene, in The Cancer Genome Atlas (TCGA) dataset (endometrioid and serous EC) {*n* = 477}, (*p* = 1, cox-regression = −17.08).

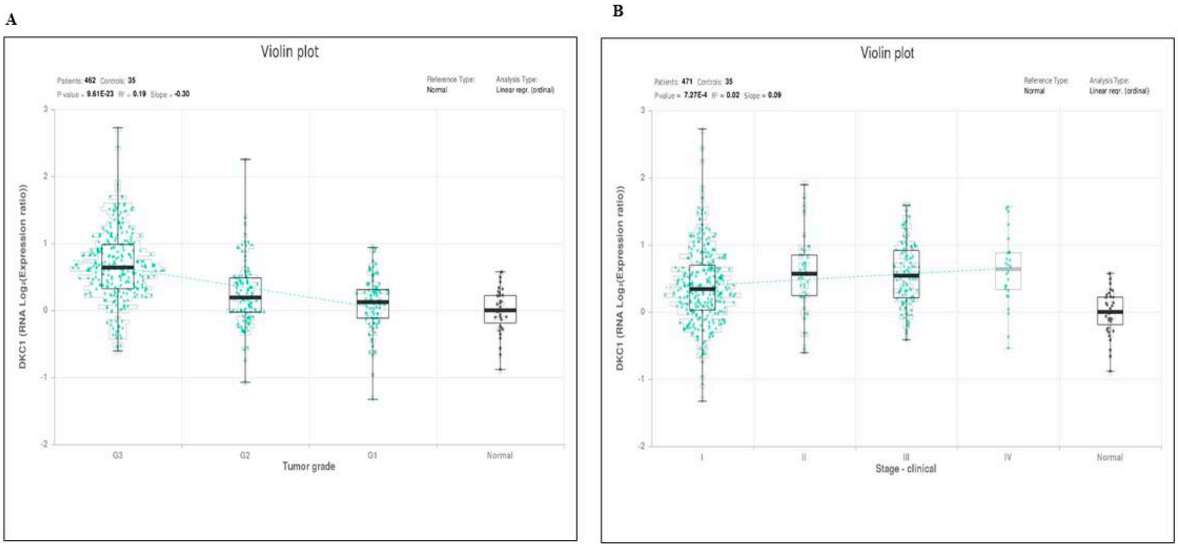

Figure S3. Violin plot demonstrating the correlation between DKC1 RNA levels with tumour grade and stage (**A**) Tumour grade {*n* = 462}, controls {*n* = 35}, ($r^2 = 0.19$, $p = 9.61 \times 10^{-23}$), analysis type linear regression, reference: normal tissue. (**B**) DKC1 RNA level correlation with endometrial cancer (EC) stage {*n* = 471}, controls {*n* =35}, ($r^2 = 0.02$, $p = 7.27 \times 10^{-4}$), analysis type linear regression, reference type is normal.

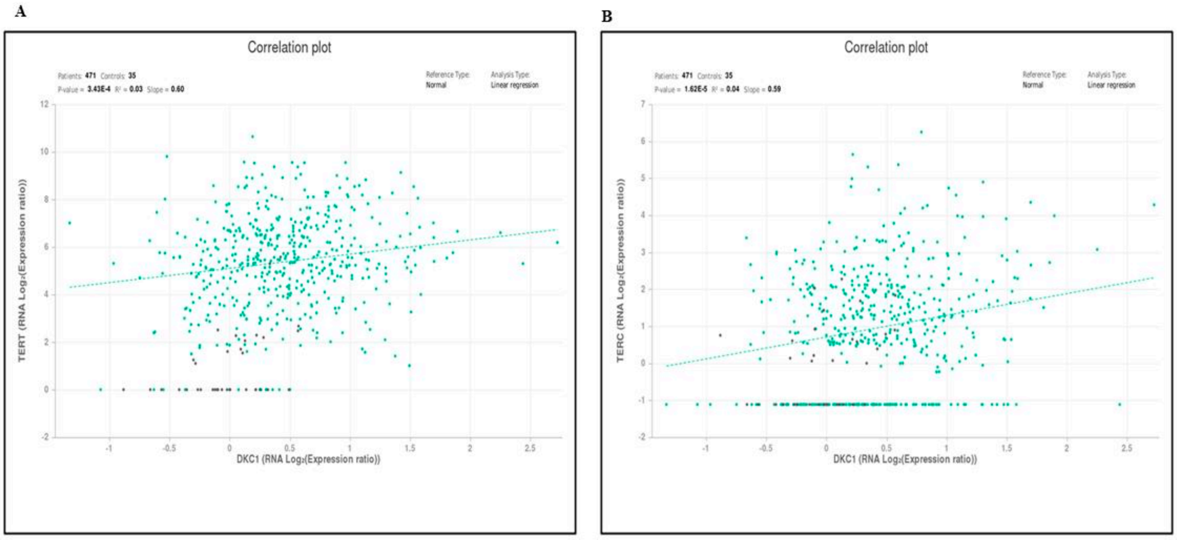

Figure S4. DKC1 RNA level correlation with TERT and TERC RNA levels in The Cancer Genome Atlas (TCGA) dataset (endometrioid and serous endometrial cancers). (**A**) Correlation with TERT RNA levels, ($r^2 = 0.03$ $p = 3.34 \times 10^{-4}$) and with (**B**) Correlation with TERC RNA levels, ($r^2 = 0.04$, $p = 1.62 \times 10^{-5}$). Patients {*n* = 471}, controls {*n* = 35}, analysis type linear regression, reference is normal control.

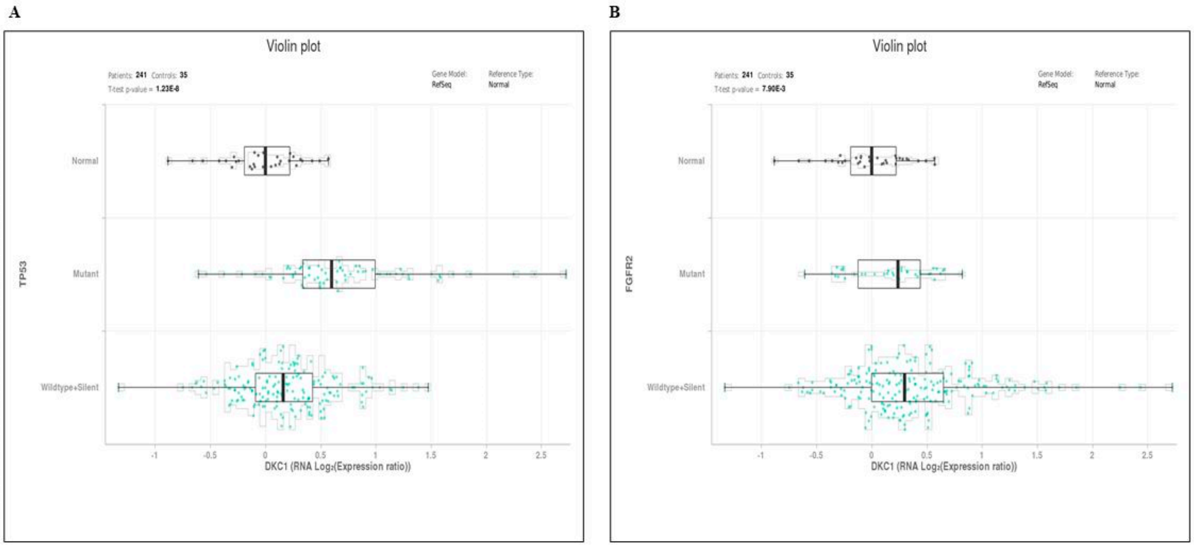

Figure S5. Violin plot shows the association between DKC1 RNA levels with the mutation status: normal, mutant or wildtype + silent of: (A) *TP53* gene, *t*-test ($p = 1.23 \times 10^{-8}$). B) FGFR2 gene, *t*-test ($p = 7.90 \times 10^{-3}$) in the Cancer Genome Atlas (TCGA) dataset (endometrioid and serous endometrial cancer). Patients ($n = 241$) and normal reference ($n = 35$).

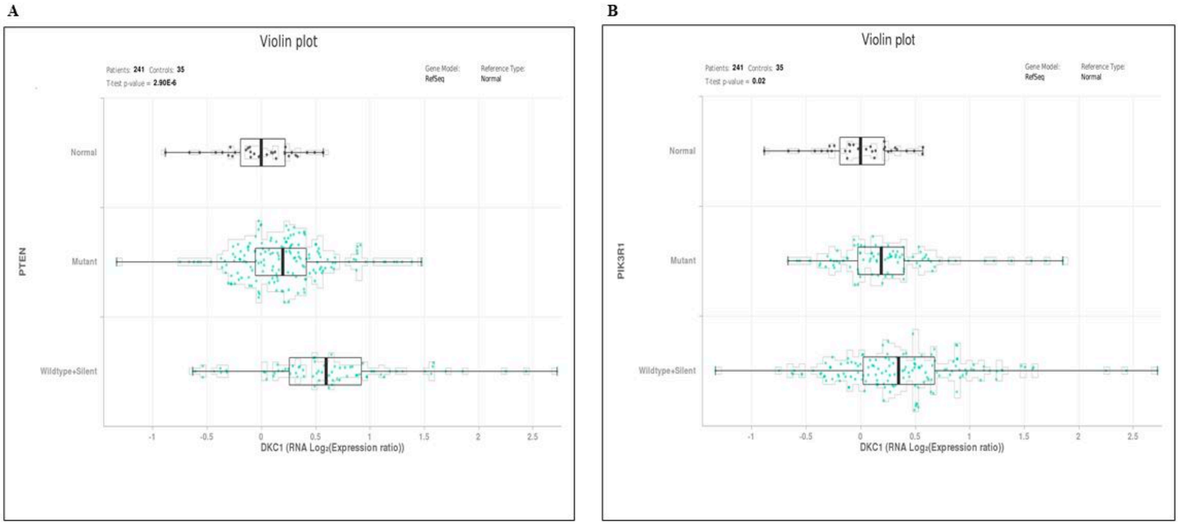

Figure S6. Violin plot shows the association between DKC1 RNA levels and mutation status: normal, mutant or wildtype + silence of (**A**) *PTEN* gene, *t*-test ($p = 2.90 \times 10^{-6}$), (**B**) *PIK3R1* gene, *t*-test ($p = 0.02$), in The Cancer Genome Atlas (TCGA) dataset (endometrioid and serous endometrial cancer), Patients ($n = 241$) and normal reference ($n = 35$).

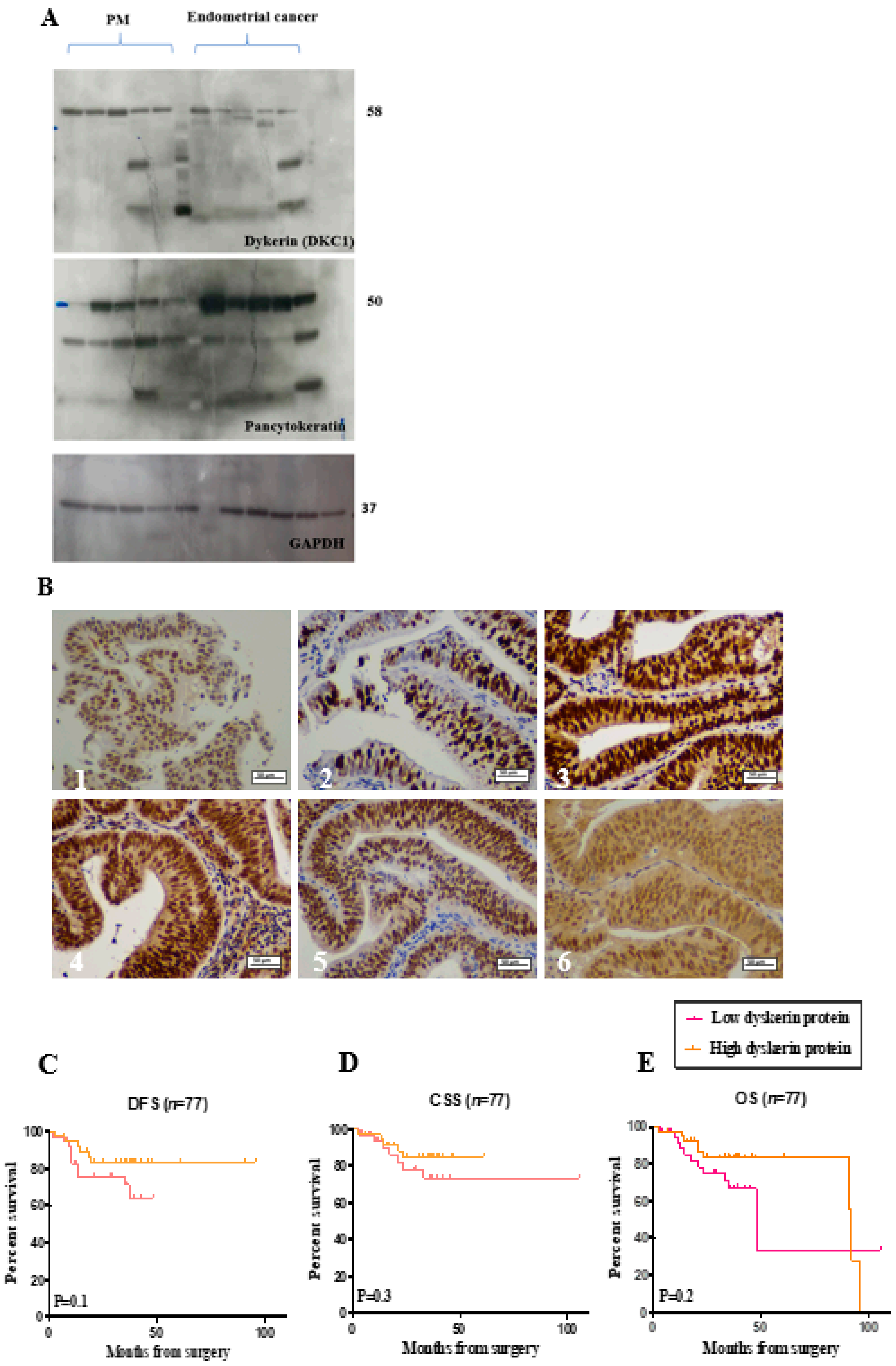

Figure S7. Whole immunoblots, dyskerin, Ki67 and steroid receptors immuno-histochemical staining and the association of dyskerin immunoscores with survival outcome. (**A**) Uncropped whole immunoblots, demonstrating dyskerin, pancytokeratin and GAPDH protein levels. (**B**) Representative microphotographs illustrating immunostaining of (1) Dyskerin, (2) Ki67, (3) ERα, (4) ERβ, (5) PR, (6) AR. Magnification 400× *g*. Scale bar 50μm in endometrial cancer (EC) samples. (**C, D, E**) Kaplan Meier survival curves for the correlation between dyskerin immunoscores and patient outcome: (**C**) Disease-free survival (DFS) median DFS is undefined for low dyskerin and high dyskerin endometrial cancer groups, HR = 2.169, 95% CI of HR (0.7999–5.882), (**D**) Cancer-specific survival (CSS); median CSS is undefined for low dyskerin and high

dyskerin endometrial cancer groups, HR = 1.762, 95% CI of HR (0.5607–5.539) and (**E**) Overall survival (OS); median OS: Low dyskerin protein = 48 months, High dyskerin protein = 92 months, Ratio of low/high dyskerin median survival = 0.5217, 95% CI of ratio (0.1074–0.9361), HR = 1.698, 95% CI of HR (0.6925–4.165) respectively in endometrioid and serous EC samples {n = 77}. Quick score of 6 was chosen as the cut-off point.

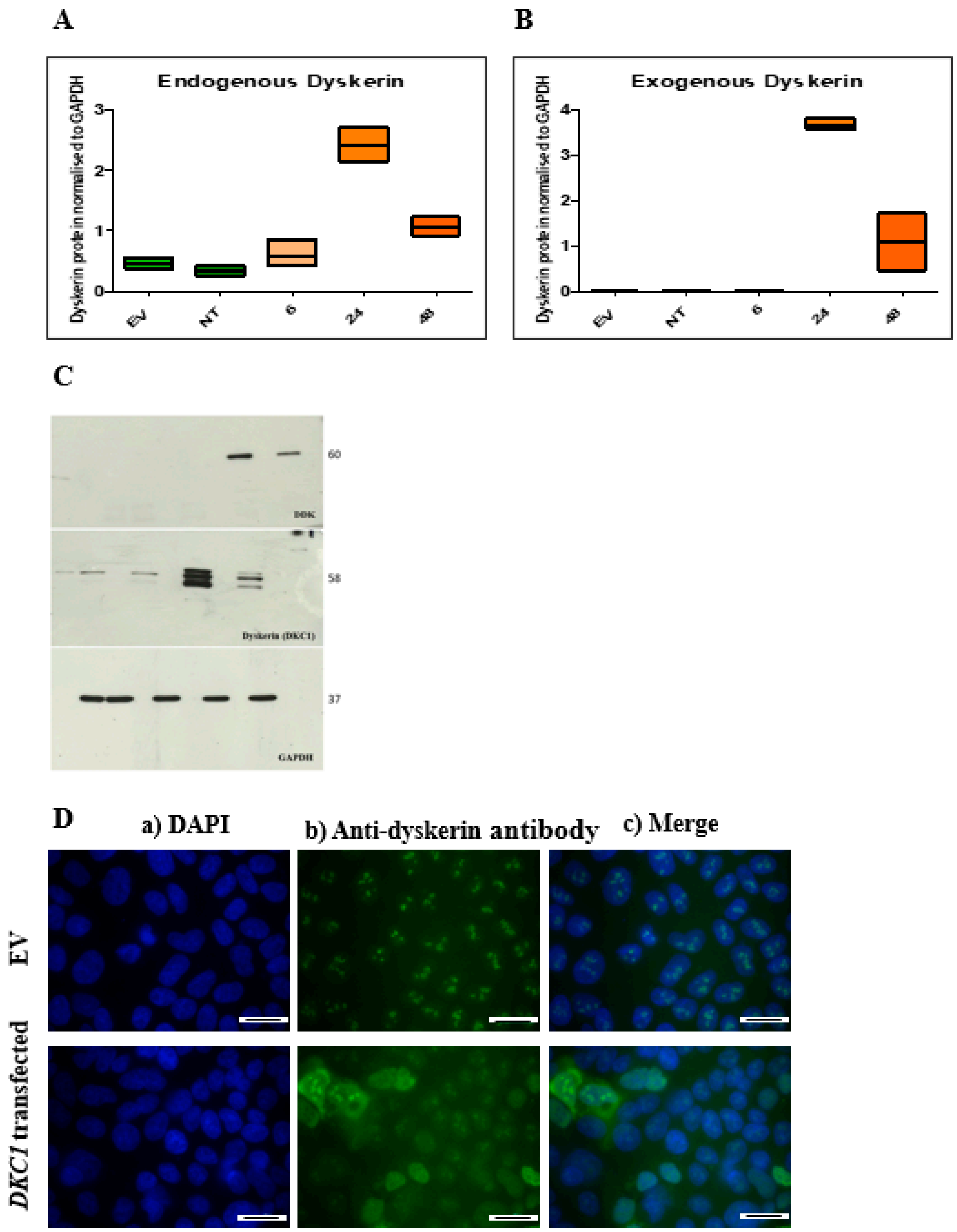

Figure S8. Transient overexpression of the *DKC1* gene in (Ishikawa) ISK cells (immunoblotting and immunofluorescence experiments) (**A**) and (**B**) Endogenous and exogenous dyskerin bands (shown in Figure 6A) were quantified using Image J. Dyskerin protein was normalised to GAPDH to ensure an equal amount of protein to be loaded in each well and Kruskal-Wallis test was used to evaluate the difference in dyskerin protein among all 3-time points of transfected cells (6, 24, 48 hours) as well as the negative controls: EV and non-transfected cells (NT). (**C**) Uncropped whole immunoblots, demonstrating DDK tag protein, dyskerin and GAPDH protein levels (**D**) Immunofluorescence staining of Ishikawa (ISK) cells,

rabbit anti-dyskerin is the primary antibody; the secondary antibodies used in this experiment was Alexa Fluor conjugated (488). Panel a) DAPI b) anti-dyskerin antibody and c) merge of a) and b). Empty vector (EV) and *DKC1* transfected ISK cells. Blue colour represents staining of cell nuclei with (DAPI), green colour represents dyskerin staining. Magnification 100x. Scale bar 100μm.

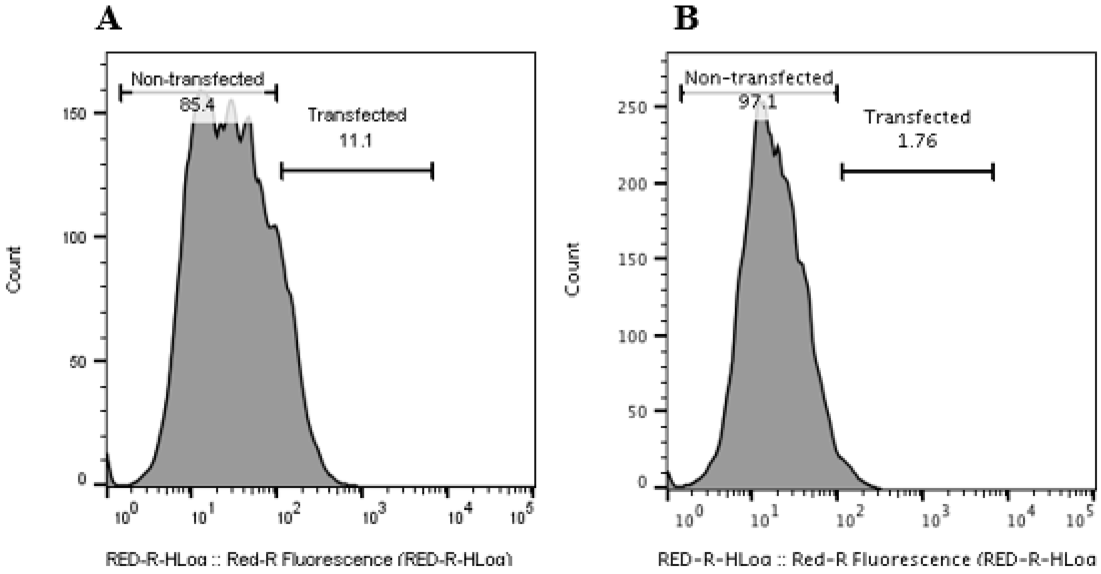

Figure S9. Negative controls used in the transient transfection experiment in Ishikawa cells. (**A**) S9 pCMV6-Entry, mammalian vector with C-terminal Myc- DDK Tag (Empty vector) (Origene technologies, USA) transfected cells. (**B**) Non-transfected cells.

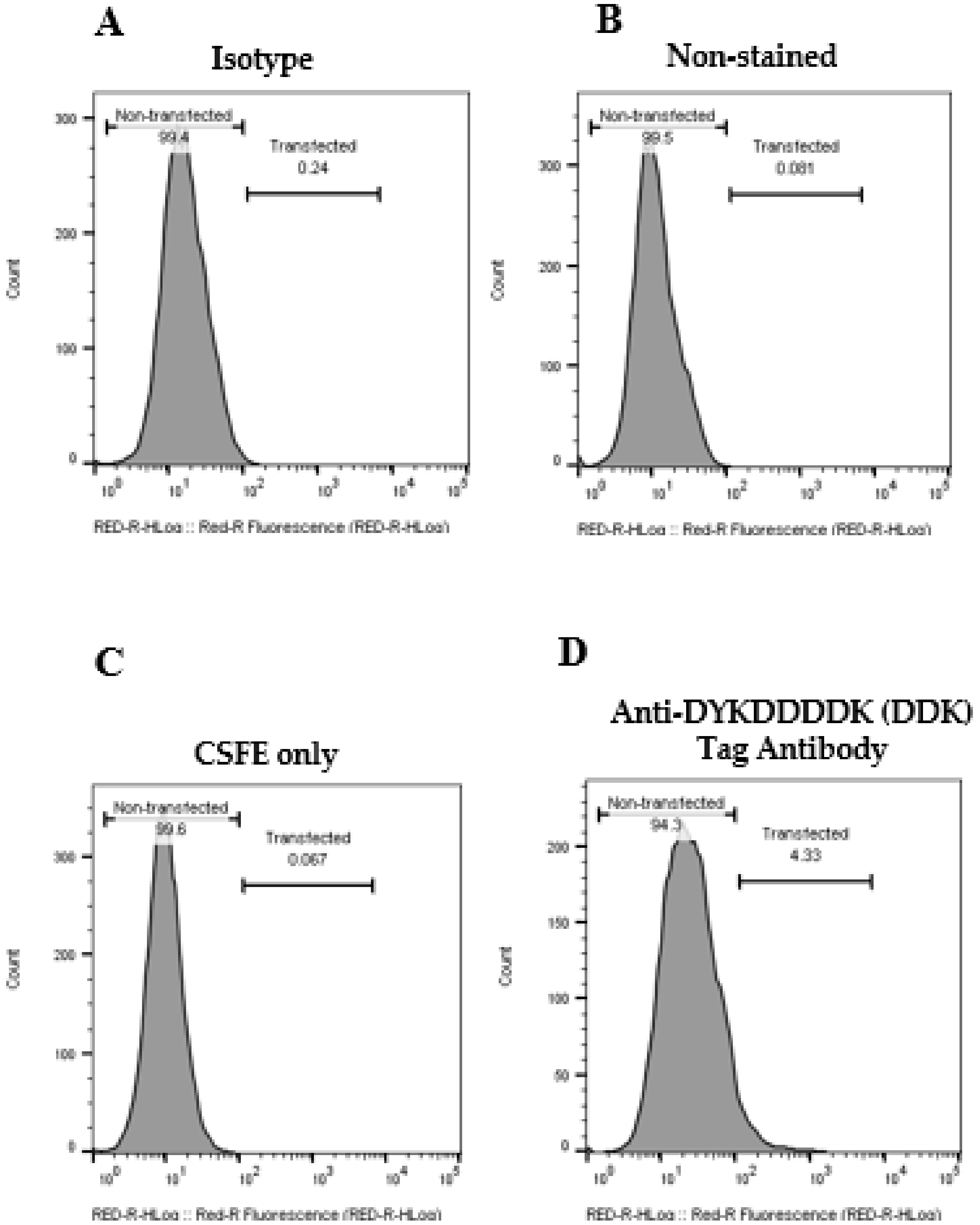

Figure S10. Negative staining controls used in the transient transfection experiment in Ishikawa (ISK) cells. DDK tagged dyskerin (*DKC1)* plasmid (Origene, USA) was used to perform transient transfection in Ishikawa cells. Flow cytometry was performed 48 hours after transfection using a Guava Easycyte flow cytometer (Millipore, Germany). Data were analysed using FlowJo v10. (**A**) Transfected Ishikawa cells stained with fluorochrome-conjugated isotype control antibody Alexa Fluor 647 (Biolegend, UK). (**B**) Non- stained DKC1 transfected cells. (**C**) Cells stained with CellTrace Carboxyfluorescein Diacetate Succinimidyl Ester (CFSE) only. (**D**) Fluorochrome-conjugated primary anti-DYKDDDDK (DDK) Tag Antibody (iFluor 647), (Genscript, USA) stained cells.

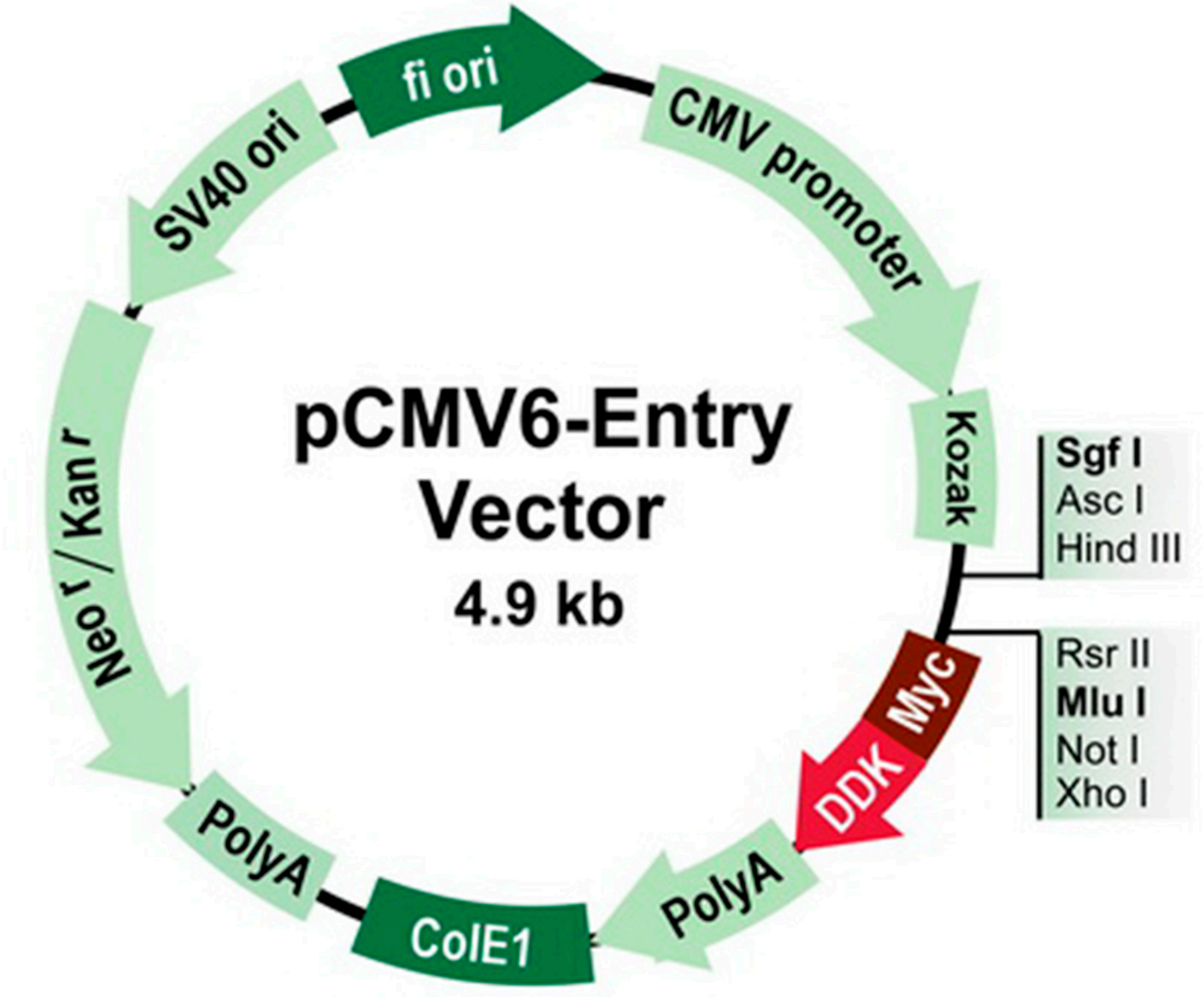

Figure S11. Map of pCMV6-Entry mammalian vector with C-terminal Myc- DDK Tag (Origene technologies, USA) used to perform transient transfection in Ishikawa cells.

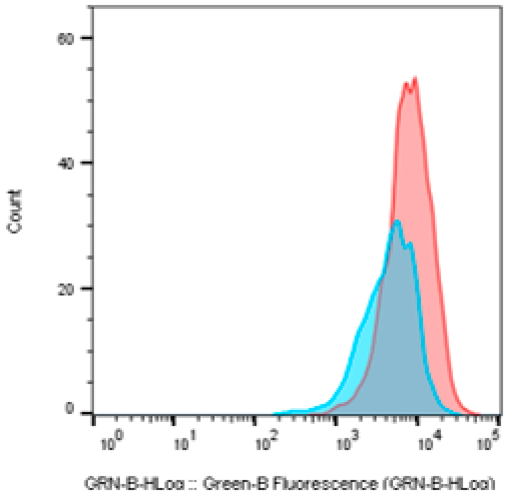

Figure S12. Cell proliferation in transfected Ishikawa (ISK) cells. The DKC1 plasmid and empty vector (EV) used were tagged with the synthetic DYKDDDDK (DDK) protein to discern the transfected cells by using an anti- DDK antibody. Cell proliferation was evaluated in DKC1 plasmid EV transfected ISK cells. The cells were stained with CellTrace Carboxyfluorescein Diacetate Succinimidyl Ester (CFSE) and fluorochrome-conjugated DDK Tag Antibody. Cells transfected with the DKC1 plasmid (red curve) and with EV (blue curve). Flow cytometry was performed 48 h after transfection using a Guava easycyte with 488-nm excitation and a 530/30-nm bandpass emission filter for CellTrace CFSE. Data were analysed using FlowJo v10 (Becton Dickinson, USA). Higher proliferation is suggested when the curve was shifted to the left.
